# Laboratory Study and Field Validation of the Performance of Salt-Storage Asphalt Mixtures

**DOI:** 10.3390/ma15196720

**Published:** 2022-09-27

**Authors:** Yangsen Cao, Xinzhou Li, Zhuangzhuang Liu, Jiarong Li, Fan Zhang, Baozeng Shan

**Affiliations:** 1 School of Highway, Chang’an University, Xi’an 710064, China; yscao@chd.edu.cn (Y.C.); lixinzhou@chd.edu.cn (X.L.); jrl@chd.edu.cn (J.L.); 2019021007@chd.edu.cn (B.S.); 2 Key Laboratory for Special Area Highway Engineering of Ministry of Education, Chang’an University, South 2nd Ring Road Middle Section, Xi’an 710064, China

**Keywords:** road engineering, salt-storage asphalt mixture, mechanical properties, surface properties, construction performance, high-elastic agents

## Abstract

The traditional method of removing ice and snow on roads carries the risk of damaging roads and the environment. In this circumstance, the technology of salt-storage asphalt pavement has gradually attracted attention. However, snow-melting salts may also have an impact on asphalt mixture performance. To explore the effect of snow-melting salts on the mechanical and surface properties of salt-storage asphalt mixtures (SSAM), SSAMs were prepared with styrene–butadiene–styrene (SBS)-modified asphalt and high-elastic asphalt (HEA) as binders and snow-melting salts as fillers. The influence of the type of asphalt binder and the content of snow-melting salt on the performance of the SSAM was preliminarily investigated through laboratory tests. The results show that the high-temperature, low-temperature, and moisture resistance performance of the SBS group SSAM decreased by 9.8–15.1%, 1.6–12.3%, and 6.3–19.4%, respectively, compared with SBS00. The higher the amount of snow-melting salt, the greater the performance drop. The three mechanical properties of the HEA group containing high-elastic agent TPS are 11.3–19.7%, 4.2–12.3%, and 4.8–13.3% higher than that of the SBS group. Even when the content of snow-melting salt is 50% or 75%, the mechanical properties of the HEA group are better than that of SBS00 without snow-melting salt. Snow-melting salt has clear advantages in improving the anti-skid performance but decreases the anti-spalling performance. The surface properties of the HEA group were also better than that of the SBS group. Considering the mechanical properties and surface properties, the comprehensive performance of the HEA group is better than that of the SBS group, and HEA50 has the best comprehensive performance. In addition, the construction performance of the SSAM has also been verified, and the production of SSAM according to the hot mix asphalt can meet the specification requirements.

## 1. Introduction

Snow and ice on the road affect traffic safety. In China, 15–35% of traffic accidents in winter are related to snow and ice [1,2]. Ice and snow removal has always caught the attention of road practitioners. Currently, the methods of road ice and snow removal mainly include the passive method and active method [3]. Passive snow removal mainly relies on traditional methods such as spreading snow-melting salts, mechanical snow removal, and manual snow removal. These three passive ice and snow removal methods are generally used in combination. Although traffic is guaranteed to a certain extent, there are problems such as untimely snow removal, the large consumption of manpower and material resources, damage to pavements, and pollution of the environment [4]. The active method mainly involves changing the pavement material or structure so that ice and snow can easily be removed from the pavement. With the development of pavement technology, the method of active ice and snow removal has gradually attracted researchers’ attention.

Active ice and snow removal technologies include elastic pavement [5,6], thermal pavement [7,8], and salt-storage pavement [9,10]. The elastic pavement technology provides the asphalt pavement with a certain elasticity by replacing the fine aggregate in the asphalt mixture with rubber particles. Under the vehicle load, the non-cooperative deformation of the ice layer and the pavement promotes ice layer rupture to achieve the purpose of deicing [11]. The structure of thermal pavements is changed to allow heating cables [12] or heat pipes [13] to be arranged inside the pavement. The purpose of melting ice and snow is achieved by employing cable heating or thermal fluid in heat pipes. Salt-storage pavement is to reduce the freezing point of water by changing the composition of asphalt mixture materials, thereby inhibiting icing. For example, snow-melting salt replaces mineral powder [14], or salt-storage aggregate replaces traditional aggregate [15]. Among the three active snow removal technologies, elastic pavement technology increases the flexibility of the pavement, its mixing process and rolling process are not yet mature, and there may also be a risk of increasing vehicle fuel consumption [3].

Thermal pavements have a high efficiency for melting ice and snow, but have the disadvantages of a high energy consumption, high cost, and complicated construction and maintenance [3]. Salt-storage pavement technology inherits the construction technology behind current pavements; this technology is favored by researchers and managers due to its low cost and successful snow-melting effect [16].

Salt-storage pavement technology originated in Europe in the 1960s, and similar research was carried out earlier in Switzerland and Germany. Verglimit, an antifreeze developed in Switzerland, reduces the freezing point of pavements to −20 °C [17]. In 1974, Austria laid salt-storage asphalt pavements on Europa Bridge [18]. Japan also introduced salt-storage technology in the 1970s, and in the 1990s Mafilon products were innovated and successfully promoted. The snow removal validity period of Mafilon can reach 5–6 years [19]. China suffered a major snowstorm in early 2008, which not only affected traffic, but also caused huge damage to societal infrastructure. In the summer of 2008, the Lanshang expressway paved the salt-storage asphalt pavement for the first time in China [18].

Some achievements have been made in the research on salt-storage pavement technology. Feng et al. [20] studied the effect of salt and freeze–thaw cycles on asphalt pavement materials, finding that salt can reduce the low-temperature performance of asphalt. When the salt content is lower than 3%, the reduction in the strength of asphalt mixtures is mainly caused by freeze–thaw cycles. When the salt content is higher than 3%, the corrosion of the salt accelerates the destruction of the asphalt mixture. Lysbakken and Lalagüe [21] studied the reliability of the BOBO 20 instrument in detecting the salt content of the pavement by testing the concentration of the salt solution, dry salt, and recrystallized salt content of the pavement. The test results indicate that the instrument is not sensitive to the content of dry salt and recrystallized salt. L. Borst and Brown [22] and Hossain et al. [23] studied the effect of pavement type on the chloride ion release rate and snow melting rate, respectively. In terms of chloride ion release rate, porous asphalt was released the fastest, followed by porous interlocking pavement, and finally porous concrete. In terms of the snow melting rate, asphalt concrete has the fastest snow melting rate, and cement concrete and interlocking concrete have similar snow melting rates. Roseen et al. [24] compared the control level of chloride ions during winter pavement maintenance between a porous asphalt mixture and densely graded asphalt mixture. Under the same ice and snow coverage and anti-skid index, the salt consumption of the porous asphalt mixture is 64–77% less than that of the densely graded asphalt mixture. Derya et al. [25] dissolved formate in a hydrophilic gel medium and then dispersed the gel in SBS polymer-modified asphalt. The anti-condensation ice results show that the modified asphalt containing formate starts to freeze 20 min later than the base asphalt at −14 °C. Over the 67-day test period, 1.07–10.8% of potassium formate was released. Zheng et al. [26] studied the long-term performance of anti-icing pavement and pointed out that snow-melting salt can improve the high-temperature performance of asphalt mixtures and reduce low-temperature performance and moisture resistance. The long-term anti-condensation ice model of pavements was established by indoor salt precipitation tests. In addition to the anti-condensation ice model, İkiz and Galip [27] studied the decay law of salt in porous pavements and cement concrete pavements with time and traffic volume, establishing a residual salt prediction model.

The above research on salt-storage pavement has promoted the development of ice and snow melting technology. The release performance of chloride ions and the performance of melting ice and snow are the functional performance of salt-storage pavement. However, the excellent mechanical properties of salt-storage pavements are fundamental for meeting service conditions. However, studies on the mechanical properties of SSAMs are limited, and some studies found that snow-melting salts can damage some properties of asphalt mixtures, such as low-temperature performance, moisture resistance, etc. [28,29,30]. Therefore, further fundamental research on the mechanical properties of SSAM needs to be further carried out. As a surface layer or wear layer, the anti-skid performance of the SSAM is also very important. At this stage, snow-melting salts are mostly chlorine-based snow-melting salts. From the perspective of mixture composition, snow-melting salt can replace traditional limestone fillers. However, the chemical properties of chlorine-based materials and alkaline limestone fillers are quite different, which may also affect the spalling resistance of asphalt mixtures, further leading to the loosening of the wear layer. In addition, most of the current research on SSAMs focuses on indoor research, and there are few reports on the verification of its field performance.

To further explore the effect of snow-melting salts on the mechanical properties and surface properties of SSAMs, SSAMs were prepared with SBS-modified asphalt and HEA as adhesives, and slow-release snow-melting salt as a limestone filler substitute. The basic mechanical properties of SSAM were studied using high-temperature performance tests, moisture resistance performance tests, and low-temperature performance tests. The surface properties of SSAMs were evaluated by anti-skid performance tests and anti-spalling performance tests. Finally, in terms of the physical project, the engineering performance and construction quality of the SSAM were tested.

## 2. Materials and Methods

### 2.1. Materials and Mix Design

#### 2.1.1. Materials

There are two kinds of asphalt that are typically used in indoor pre-studies: SBS I-D modified asphalt and HEA. HEA is prepared by adding TPS high-elastic agent into SBS I-D modified asphalt. The technical indicators of the two asphalts are shown in Table 1. The coarse aggregate and fine aggregate are limestones from a quarry in Liquan, Xi’an, China. Aggregate technical indicators are shown in Table 2. The snow-melting salt in the SSAM was added in the form of filler, so there are two types of fillers used in the test: conventional limestone filler and snow-melting salt filler. The technical indicators of the two fillers are shown in Table 3. Stone mastic asphalt (SMA) with a good durability is selected for the wear layer, and the technical indicators of lignin fibers are shown in Table 4.

#### 2.1.2. Mix Design

**Figure 1:**
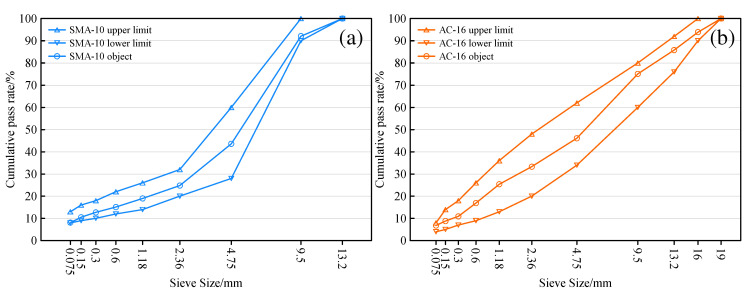
Gradation curves of SMA-10 and AC-16. (**a**) Gradation curve of SMA-10; (**b**) Gradation curve of AC-16.

The gradation type of the wear layer in this study is SMA-10. To ensure that the wear layer is applicable in follow-up research, the suspension compact asphalt mixture AC-16 is selected as the lower bearing layer of SMA-10. The gradation curves of SMA-10 and AC-16 are shown in Figure 1. Snow-melting salt is added to asphalt mixes as fillers. Due to the large difference in density between the snow-melting salt and the limestone filler, the equal volume replacement method was used to replace the limestone filler with snow-melting salt. SMA-10 asphalt mixtures are named in terms of asphalt type and snow-melting salt replacement ratio. There are seven kinds of SMA-10 asphalt mixture, namely SBS00, SBS50, SBS75, SBS100, HEA50, HEA75, and HEA100. The asphalt aggregate ratio of SMA-10 and AC-16 was determined using the Marshall test. The optimal asphalt aggregate ratio was finally determined to be 6.2% for SMA-10 and 4.2% for AC-16.

### 2.2. Laboratory Test Methods

#### 2.2.1. High-Temperature Performance Test

To truly reflect the anti-rutting performance of SSAM at a high temperature, SMA-10 + AC-16 composite specimens were prepared for rutting tests. The size of the specimen is 300 × 300 × 50 mm^3^, of which the thickness of SMA-10 is 20 mm, and the thickness of AC-16 is 30 mm. The specimen was compacted twice: first, the cushion layer AC-16 was formed, and then the SMA-10 was rolled on the cushion layer. The test was carried out according to the specification [31], the test temperature was 60 °C, and the wheel pressure was 0.7 MPa. Rutting at 45 min and 60 min was recorded, and the dynamic stability was calculated accordingly. The average value of three sets of tests was taken as the final dynamic stability to evaluate the high-temperature performance of different salt-storage wear layers.

#### 2.2.2. Moisture Resistance Performance Test

As an alkaline aggregate, limestone filler has a good adhesion to asphalt; therefore, asphalt is a better coating for the aggregate. In the salt-storage wear layer, since the filler is replaced with slow-release snow-melting salt, even if the surface layer of the snow-melting salt is wrapped with lipophilic components, its interaction with asphalt is still not as good as that of the limestone filler, which may affect the moisture resistance performance of salt-storage wear layer.

The immersion Marshall test was used to test the stability of standard Marshall specimens before and after immersion and compare the changes in stability before and after immersion. First, standard Marshall specimens were prepared and divided into two groups: one group was placed in a 60 °C constant temperature water tank for half an hour, and the other group was placed in a 60 °C water tank for 48 h. After that, the stability of each group of specimens was measured, and the residual stability ratio was calculated.

The freeze–thaw splitting test was also needed to divide the mixture specimens into two groups. One group is to measure the splitting strength at room temperature, and the other group requires the specimens to be saturated with water under vacuum conditions, and then the specimens are frozen at −18 °C. After that, the specimens were placed in a constant temperature water tank at 60 °C for 24 h, and the splitting strength of the specimens was finally determined. The moisture resistance performance was evaluated by the ratio of splitting strength of two groups of specimens under freeze–thaw and unfreeze–thaw conditions. The asphalt film on the aggregate surface is more susceptible to the effects of water at low temperatures, so it peeled off from the aggregate surface. Therefore, compared with the water immersion Marshall test, the freeze–thaw splitting test can better reflect the moisture resistance of the salt-storage wear layer.

#### 2.2.3. Low-Temperature Performance Test

Winter is the season when salt-storage asphalt pavements are most required, so these pavements must have a sufficient low-temperature crack resistance. The low-temperature cracking of asphalt pavement mainly occurs in two different ways: material shrinkage cracking and fatigue cracking. In this paper, the low-temperature shrinkage performance of different types of salt-storage wear layers is studied using a low-temperature bending test.

The size of the specimen is 30 × 35 × 250 mm^3^. Loading was carried out by a three-point loading method with a span of 200 mm until the specimen broke. The loading rate was 50 mm/min, and the experiments were carried out at −10 °C. Low-temperature performance is usually evaluated by a low-temperature failure strain after the test. In addition to the low-temperature failure strain, the fracture energy density was also used to evaluate the low-temperature crack resistance of the mixture. Strain energy density, as a kind of energy stored in a unit volume, can reflect the fracture ability of a material.

#### 2.2.4. Surface Anti-Skid Performance Test

The friction between the tires and the pavement is an important factor to ensure the safe driving of the vehicle, especially in winter. Therefore, it is necessary to conduct a skid resistance test on the wear layer. This time, the pendulum tribometer BM-III was used to determine the anti-skid properties of different types of salt-storage wear layers. The test specimens used in the test are the same as those used in the rutting test.

#### 2.2.5. Surface Spalling Resistance Test

The spalling resistance of the wear layer largely depends on the adhesion between the asphalt and the aggregate. Conventional tests for measuring adhesion include the boiling method, water immersion method, etc. [31], and these tests are greatly affected by subjective factors. In this study, the Schellenberg binder drainage test and the Cantabro test were used to measure the adhesion of the salt-storage wear layer. The Schellenberg binder drainage test can also be used to judge whether there is an excess of free asphalt in the mixture, and to verify the optimal asphalt aggregate ratio of the mixture. Referring to the specification [31], the Schellenberg binder drainage test was measured using a beaker. A specified amount of asphalt mixture was weighed and placed in a beaker, and then the beaker was kept in an oven at 185 °C for 60 min. Afterward, the asphalt mixture was poured out, the mass of the residual asphalt on the wall of the beaker was weighed, and the asphalt loss was calculated.

During the Cantabro test, the prepared Marshall specimens were placed in a constant temperature water tank at 20 °C for 20 h. Then, the specimen was placed in the Los Angeles abrasion testing machine for 300 revolutions. The mass loss of the specimen after the test is the loss rate of the Cantabro test.

### 2.3. Field Performance Verification of Salt-Storage Wear Layer

#### 2.3.1. Project Overview

Xi’an is located in the Guanzhong Plain, which is hot in summer and cold in winter. The pavement surface can easily freeze in winter, creating a great hidden danger to traffic safety. Snow removal methods such as sprinkling snow-melting salt and mechanical cleaning will not only corrode pavement structures and damage the surrounding environment, but also consume a lot of manpower and material resources. To eliminate road safety hazards and reduce the resources required for ice and snow removal, on 30 June 2019, an experimental road with a length of 520 m, intended for snow-melting tests, was laid on the Jinghe Bridge of National Highway 310 based on the indoor pre-study. The implementation of the project provides a reference for the construction of pavements that can automatically melt snow in the Guanzhong area.

#### 2.3.2. Main Material Parameters

Slightly different from the indoor pre-study, the asphalt used in the project is SBS I-C-modified asphalt. The technical indicators are shown in Table 5. The coarse and fine aggregates are all limestone from Xinhe, Xi’an, China. The aggregate density of each grade is shown in Table 6, and the other indicators meet the requirements of the specification. The fillers are slow-release snow-melting salts, and the replacement rate of limestone filler is 100%.

#### 2.3.3. Mix Design for Construction

**Figure 2:**
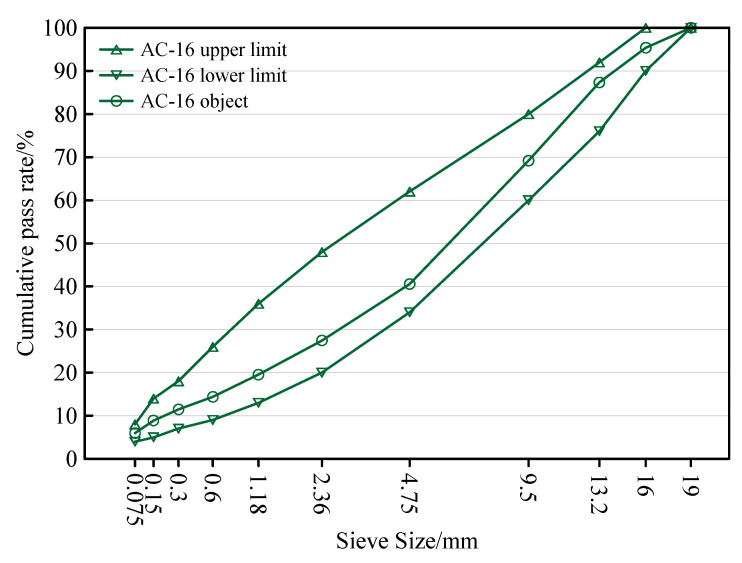
AC-16 grading curve for construction.

In the early stage, the performance of the SMA-10 salt-storage wear layer was studied indoors. For the consideration of construction costs, the project directly selected AC-16 as the wear layer of the pavement, that is, the upper layer. There are five stone silos in the mixing station, corresponding to limestone aggregates with particle sizes of 0–3, 3–6, 6–11, 11–17, and 17–22 mm, respectively. The gradation curve used for production is shown in Figure 2. Five groups of asphalt aggregate ratios of 3.8%, 4.3%, 4.8%, 5.3%, and 5.8% were proposed to determine the optimal asphalt aggregate ratio. The results of the Marshall test are shown in Table 7. The optimal asphalt aggregate ratio determined by calculation is 4.135%, and then the asphalt aggregate ratio is adjusted to 4.2%. The performance indicators of the AC-16 asphalt mixture under the best asphalt aggregate ratio are also shown in Table 7.

## 3. Results and Discussion

### 3.1. Mechanical Properties

#### 3.1.1. High-Temperature Stability

**Figure 3:**
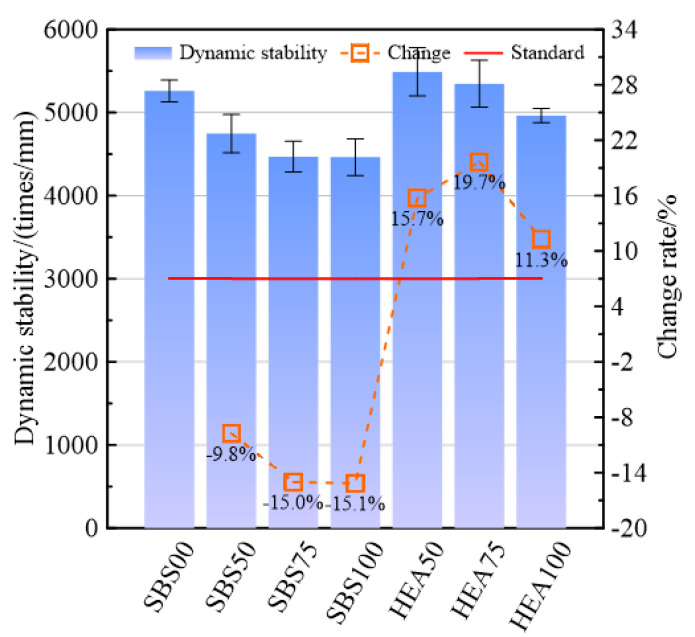
High-temperature stability performance of salt-storage asphalt wear layer.

Figure 3 shows the high-temperature performance of different types of SSAMs. Comparing the salt-storing asphalt mixture in the SBS group with SBS00 without snow-melting salt, it can be seen that the snow-melting salt reduces the high-temperature performance of the asphalt mixture. The dynamic stability of SBS50, SBS75, and SBS100 was 9.8%, 15.0%, and 15.1% lower than that of SBS00, respectively. This may be because the interaction between snow-melting salt and asphalt is not as good as the interaction between alkaline limestone filler and asphalt [33,34], which leads to poor adhesion between asphalt mortar and aggregate, and the shear resistance of SSAM decreases at high temperatures. When the content of snow-melting salt is the same, the high-temperature performance of SSAM in the HEA group is better than that in the SBS group. For example, when the replacement rates of snow-melting salt are 50%, 75%, and 100%, the dynamic stability of the HEA group is 15.7%, 19.7%, and 11.3% higher than those in the SBS group, respectively. This indicates HEA can significantly improve the high-temperature performance of the salt-storage wear layer. As a highly elastic polymer, the network structure formed by TPS after swelling in SBS-modified asphalt increases the elasticity of the asphalt [5], which may improve the high-temperature performance of the asphalt mixture. Whether in the SBS group or the HEA group, the snow-melting salt adversely affects the rutting resistance, and the higher the snow-melting salt content, the greater the decrease in the high-temperature performance. Compared with SBS00, the dynamic stability of HEA50 and HEA70 is even higher than that of SBS00. This shows that HEA can compensate for the negative impact of snow-melting salt on high-temperature performance to a certain extent. Among the six SSAMs, the dynamic stability of SBS75 and SBS100 with lower dynamic stability are 4469 and 4462 times/mm, respectively. The highest dynamic stability is HEA50. Even the SBS100 with the lowest dynamic stability, its high-temperature performance can meet the specification requirements of 3000 times/mm.

#### 3.1.2. Moisture Resistance

**Figure 4:**
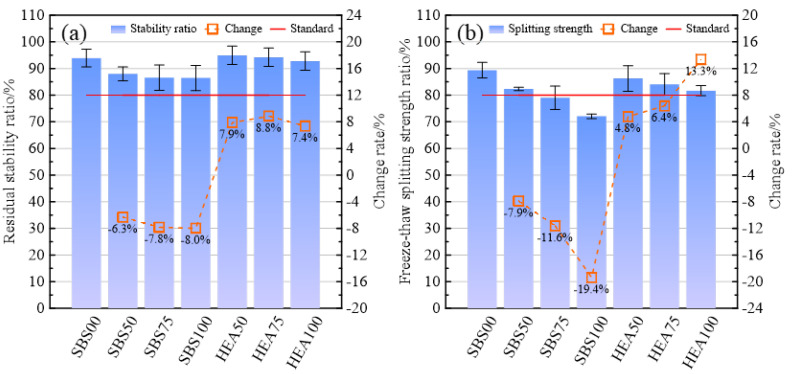
Moisture resistance performance of SSAM. (**a**) Residual stability; (**b**) splitting strength ratio.

The results of the immersion Marshall test and the freeze–thaw split test are shown in Figure 4a,b, respectively. In Figure 4a, Compared with SBS00, the residual stability of SBS50, SBS75 and SBS100 decreased by 6.3%, 7.8% and 8.0%, respectively. Snow-melting salt is not good for moisture resistance. Snow-melting salts can lead to the reduction in structural asphalt in the asphalt, which in turn reduces the integrity of the asphalt mixture, resulting in a decrease in moisture resistance [16]. In addition, after the snow-melting salt is dissolved in water, the micropores of the asphalt mixture increase, and the water is more likely to penetrate into the interior of the asphalt mixture [33]. This may also be one of the reasons for the decrease in the residual stability ratio. In the HEA group, the addition of the high-elastic agent TPS significantly improved the residual stability ratio. HEA50, HEA75 and HEA100 were 7.9%, 8.8% and 7.4% higher than SBS50, SBS75 and SBS100, respectively. This shows that TPS increased the bond between the asphalt and the aggregate. The viscosity of HEA is higher than that of SBS-modified asphalt in Table 1, which supports this view. For two groups of SSAM, the higher the replacement rate of snow-melting salt, the greater the decrease in residual stability. When the replacement rate of snow-melting salt is lower than 75%, the moisture resistance of HEA50 and HEA75 is even higher than that of SBS00 without snow-melting salt. The high viscosity of HEA compensates for the weaker adhesion between snow-melting salt and asphalt, making the asphalt mixture more resistant to moisture damage. Among the SSAMs, the lowest and highest residual stability ratios are SBS100 and HEA50, respectively. Although snow-melting salts were detrimental to the residual stability ratios, the residual stability ratios of all SSAMs were higher than the 80% required by the specification.

In Figure 4b, the freeze–thaw splitting strength ratio of the SSAM in the SBS group is lower than that of the SBS00, and the decrease of the SBS100 even reaches 19.4%. In addition, to the void structure left by the precipitation of snow-melting salt and the reduction in asphalt cohesion by snow-melting salt, the corrosion effect of the salt solution may also be one of the reasons for the decline of moisture resistance [26,34]. Compared with the SBS group, the splitting strength ratio of the HEA group was increased to varying degrees, and the most obvious increase was HEA100, which was 13.3% higher than that of SBS100. The better viscosity and low-temperature properties of HEA are responsible for the improved splitting strength ratio. Similar to Figure 4a, for both the SBS group and the HEA group, as the content of snow-melting salt increases, the freeze–thaw splitting strength ratio of the mixture decreases linearly, and this is especially evident in the SBS group. Although HEA improves the moisture resistance of SSAM to a certain extent, the freeze–thaw splitting strength ratio of all SSAMs is lower than that of SBS00. It shows that the improvement effect of HEA is limited. Even the splitting strength ratio of HEA50 with the highest splitting strength ratio is about 3.0% lower than that of SBS00. According to the specification requirements [35], the freeze–thaw splitting strength ratio of SMA using modified asphalt should not be lower than 80%. The freeze–thaw splitting strength ratio of SSAM using HEA can meet this requirement, but SBS75 and SBS100 do not meet the requirement. The content of snow-melting salt should not be too much, especially when using SBS-modified asphalt.

#### 3.1.3. Low-Temperature Crack Resistance

**Figure 5:**
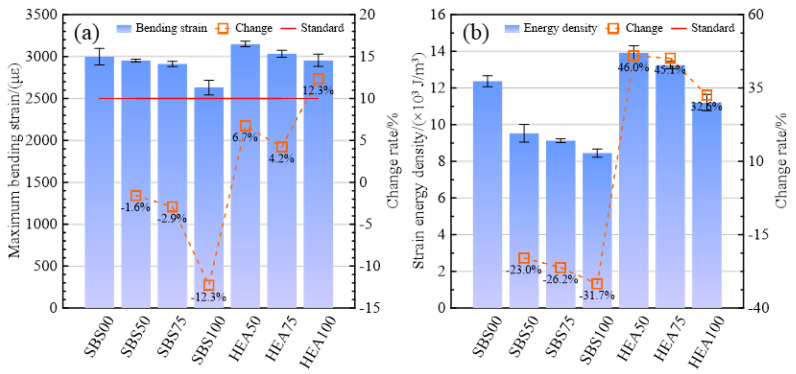
Low-temperature performance index of SSAM. (**a**) Maximum tensile strain; (**b**) strain energy density.

Figure 5a shows the tensile strain of the SSAM when it fails in bending. The larger the tensile strain, the higher the flexibility and crack resistance of the mixture at low temperatures. In the asphalt mixture of the SBS group, the maximum tensile strains of salt-asphalt mixtures are lower than SBS00. The maximum tensile strain decreased by 12.3% after all the limestone fillers were replaced with snow-melting salt. After using HEA high-elastic modified asphalt, the maximum tensile strain of the HEA group significantly increased compared with the SBS group. Compared with the SBS group, HEA50, HEA75, and HEA100 increased by 6.7%, 4.2%, and 12.3%, respectively. This is because HEA has better flexibility than SBS-modified asphalt at low temperatures, which is illustrated by the higher ductility of HEA than SBS-modified asphalt in Table 1. Regardless of the HEA group or the SBS group, the maximum tensile strain decreased with the snow-melting salt. The poor adhesion between snow-melting salt and asphalt may be the main reason for this problem. In addition, the snow-melting salt is mainly distributed in the asphalt, and its dispersibility characteristics may reduce the continuity of the asphalt on the fracture surface [20,36], and then reduce the toughness of the asphalt. Under the condition that the replacement rate does not exceed 75%, the tensile strain of the HEA group is higher than that of SBS00. Even with all limestone fillers replaced, the maximum tensile strain of HEA100 is only 1.5% lower than that of SBS00. HEA compensates for the adverse effects of snow-melting salts. Among the six SSAMs, HEA50 has the best low-temperature crack resistance, and SBS100 is the most prone to cracking. Nevertheless, the tensile strains of all SSAMs meet the specification requirements.

The strain energy densities of different types of salt-storage wear layers at −10 °C are shown in Figure 5b. The higher the strain energy density, the higher the energy required for the material to fracture, and the less likely it will fracture. The strain energy density of the SSAM in the SBS group was lower than that in the SBS00 group, and the strain energy density in the HEA group was much higher than that in the SBS group. Therefore, it can be concluded that snow-melting salt and HEA have negative and positive effects on the low-temperature crack resistance of salt-storing asphalt mixtures, respectively. The strain energy density of HEA50 and HEA75 is higher than that of SBS00, which is similar to Figure 5a. In addition, HEA50 has the highest strain energy density and SBS100 has the lowest strain energy density in the SSAM. Highly elastic asphalt is beneficial for promoting the application of salt-storage materials.

### 3.2. Surface Properties

#### 3.2.1. Surface Anti-Skid Performance

**Figure 6:**
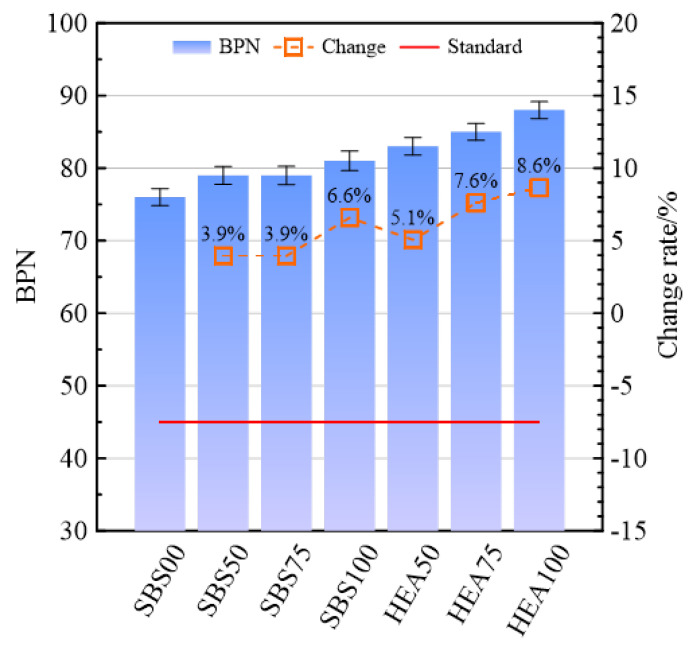
Anti-skid index of SSAM.

As a surface functional layer, the SSAM must meet the anti-skid requirements to ensure driving safety while meeting the conventional mechanical properties. The anti-skid performance of different SSAMs is shown in Figure 6. The British pendulum number (BPN) of the SSAM in the SBS group was higher than that of the SBS00, and the BPN of the SBS100 increased most significantly by 6.6%. This shows that the snow-melting salt is beneficial in improving the anti-skid performance. There may be two reasons, one is that the snow-melting salt particles are coarser than the limestone ore powder [9,16]. Snow-melting salt may improve the surface roughness of salt-storage asphalt [37]. Second, before testing BPN, the sprayed water may have caused the precipitation of surface salt, leaving micro-pores that make the surface of the salt-storage asphalt rougher. Under the same replacement amount of snow-melting salt, the BPN value of the asphalt mixture in the HEA group is higher than that in the SBS group. For example, the BPN value of HEA100 is 8.6% higher than that of SBS100. TPS increases the elasticity of asphalt and improves the viscosity of asphalt; therefore, high-elastic asphalt can better improve the anti-skid ability of pavements during winter. With the increase in the replacement amount of snow-melting salt, the BPN of the two groups of SSAMs gradually increased, indicating that the snow-melting salt has an advantage over the limestone filler in improving the anti-skid performance of the mixture. Among the six SSAMs, the best anti-skid performance is HEA100. Although SBS50 has the worst skid resistance, it still meets the specification requirements.

#### 3.2.2. Surface Spalling Resistance

**Figure 7:**
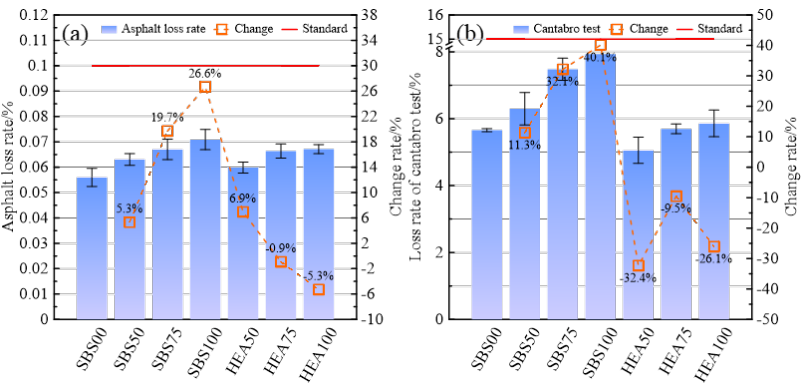
Anti-spalling index of SSAM. (**a**) Asphalt loss rate; (**b**) Cantabro loss rate.

As shown in Figure 7a, the asphalt loss of the SSAM in the SBS group is higher than that in the SBS00. Among them, the asphalt loss of SBS100 is 26.6% higher than that of SBS00. This may be because the snow-melting salt increases the free asphalt in the asphalt mixture [16]. The asphalt loss exhibited by the HEA group was different from that of the SBS group, which may be because TPS increased the high-temperature performance of SBS-modified asphalt. This is consistent with the penetration and softening points of HEA in Table 1 being lower and higher than that of SBS-modified asphalt, respectively. With the increase in the replacement rate of snow-melting salt, the quality of asphalt adhering to the beaker increases, especially in the SBS group. Therefore, the content of free asphalt in the mixture may increase with the content of snow-melting salt. This may be because the addition of snow-melting salt reduces the optimum asphalt aggregate ratio of the mix. However, the fillers used in determining the optimal asphalt aggregate ratio are all limestone fillers, and the optimal asphalt aggregate ratio may slightly change after the snow-melting salt replaced part of the limestone filler. The lack of adjustment of the optimal asphalt aggregate ratio resulted in a larger asphalt loss of the specimens with snow-melting salt than that without snow-melting salts [38]. Compared with the SBS00 group, the SSAM in the HEA group has a higher asphalt loss rate. Therefore, although HEA is helpful to improve asphalt bonding, the gain effect of HEA on asphalt cannot completely cover the negative effect of snow-melting salt. In the SSAM, the least asphalt loss is HEA50, and the largest one is SBS100. Since the maximum asphalt loss required by the specification is 0.1%, the free asphalt content of the various SSAMs is satisfactory. This indirectly shows that the anti-spalling performance of the SSAM meets the requirements.

In Figure 7b, the mass loss of the SSAM in the SBS group was significantly higher than that in the SBS00 group. The mass loss of SBS50, SBS75, and SBS100 is 11.3%, 32.1%, and 40.1% higher than that of SBS00, respectively. This may be because the coating effect of the mortar formed by asphalt and snow-melting salt may be weak, which cannot accurately ensure the adhesion of asphalt and aggregates, reducing the integrity of the mixture. Compared with the SBS group, the HEA group had a more significant decrease trend. The high viscosity and high elasticity of HEA can improve the adhesion of asphalt mortar. Whether it is the HEA group or the SBS group, with the increase in the replacement rate of snow-melting salts, the loss of mass also tended to increase. Snow-melting salt is not good for spalling resistance of SSAM. The mass loss of HEA50 is lower than that of SBS00, and HEA75 is on par with SBS00. HEA100 is higher than SBS00, which may be caused by the change in the asphalt-filler ratio [39]. HEA improves the spalling resistance of SSAM to a certain extent. In the specification for SMA using modified asphalt, the loss rate in the Cantabro test should not exceed 15% [35]. Therefore, the anti-loose performance of the SSAM meets the requirements.

### 3.3. Determination of Optimal Dosage of Snow-Melting Salt

From the analysis in Section 3.1 and Section 3.2, it can be seen that the snow-melting salt is unfavorable to the mechanical properties of asphalt mixtures, including high-temperature performance, moisture resistance, and low-temperature performance. In terms of surface properties, snow-melting salt is beneficial to the anti-skid performance of the SSAM, but at the same time harms the anti-spalling performance. To determine the optimal dosage of snow-melting salt, the influence of snow-melting salt on mechanical properties and surface properties should be comprehensively considered. Here, the performance of various SSAMs is comprehensively evaluated by the grey target decision [40]. The indicators used in the evaluation are dynamic stability, residual stability ratio, freeze–thaw splitting strength ratio, maximum flexural–tensile strain, strain energy density, BPN, asphalt loss, and Cantabro loss. The raw data of the 8 indicators are shown in Table 8.

Among the eight indicators, except for the two economic indicators of asphalt loss and Cantabro loss, the other six indicators are benefit indicators. The lower the economic index, the better the performance of the asphalt mixture, while the benefit index is the opposite. To evaluate the comprehensive performance of SSAM, it is necessary to normalize each index [41]. The normalized values of each index are shown in Table 9. After normalization, the optimal data in each index is 1. If the data of an index are closer to 1, then the performance represented by this index is better. Here, [1,1,1,1,1,1,1,1] is used as the target vector to represent the best overall performance. The closer the distance of the vector composed of the comprehensive performance of various asphalt mixtures to the target vector, the better the comprehensive performance of the asphalt mixture.

The distances between the comprehensive performance of the seven asphalt mixtures and the target vector are shown in Table 10. If the snow melting potential is not considered, the comprehensive performance of the seven asphalt mixtures is HEA50, HEA75, SBS00, HEA100, SBS50, SBS75, and SBS100 in descending order. It can be seen that the comprehensive performance of the HEA group is better than that of the SBS group, and with the increase of snow-melting salt content, the comprehensive performance of SSAM decreases. Based on the current results, it is suggested that HEA is used as the cement for SSAM, and the content of snow-melting salt should be 50%. In practical applications, the amount of snow-melting salt must be determined by the snow-melting performance.

### 3.4. On-Site Construction Performance

#### 3.4.1. Performance Verification of Salt-Storage Wear Layer before Construction

- High-temperature performance verification

Referring to the specification [35], the climate zone where Xi’an is located is 1–3. A rutting tester was used to form three standard rutting plate specimens with a thickness of 5 cm. The rutting plate specimens were kept in an environment of 60 °C for 7 h, the rutting test was then carried out, and the tire ground pressure was 0.7 MPa. The dynamic stability data of the rutting plate are shown in Table 11. The average dynamic stability is 6368.6 times/mm, which is much higher than the maximum deformation resistance requirement of 2800 times/mm for the modified asphalt mixture in the specification.

- Moisture resistance verification

The moisture resistance test was carried out on Marshall specimens prepared from SSAMs. A group of specimens was kept at a constant temperature in a water tank at 60 °C for 48 h, and then the stability was measured. One group was kept at a constant temperature in a water tank at 60 °C for 30 min to test its stability. The stability test results are shown in Table 12. The residual stability is 87.84%, which meets the specification requirements of 80%.

- Low-temperature performance verification

According to the specification requirements [35], the flexural and tensile strain of the modified asphalt mixture should not be less than 2500 με at low-temperature failure. In Table 13, the average failure strain of the salt-storage asphalt wear layer is 2668 με, which is slightly higher than the specification requirements, so the SSAM used in the project can meet the requirements of crack resistance in winter.

- Water permeability verification

In engineering applications, in addition to testing the conventional mechanical properties, it is also necessary to verify the water permeability of the mixture. First, the rutting plate specimen was formed with SSAM, and the annular sealing area was smeared on the surface of the rutting plate with grease. The timer was started when the water in the graduated cylinder decreased to 100 mL. The scale of the water in the measuring bucket was read every 60 s. Due to the slow falling speed of the water, the amount of water seepage at 3 min was measured. The permeability coefficient is shown in Table 14. The average value of the permeability coefficient is 44.7 mL/min, which is far less than the 120 mL/min required by the specification, so the water permeability of the SSAM meets the specification requirements.

#### 3.4.2. Production and Construction of Salt-Storage Wear Layer

- Dosing of snow-melting salt

**Figure 8:**
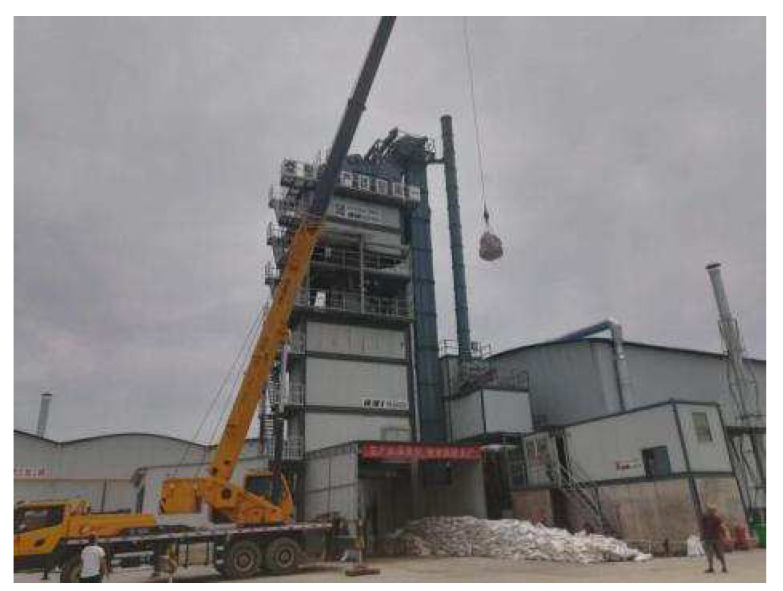
Hoisting the snow-melting salt into the mixing plant.

The asphalt mixing machine is HLB4000. The mixing method of snow-melting salt is the external mixing method, and the snow-melting salt required for one mixing was added to the mixture by temporary hoisting (Figure 8). The amount of each material required for one mixing is shown in Table 15.

- Mixing and transport

The manufacturing process of SSAM is generally carried out following the production process of ordinary asphalt mixture: raw material preparation, heating, stone consumption measurement, dry mixing, asphalt spraying and mixing for 10 s, adding snow-melting salt and mixing for more than 40 s, and discharging. The process temperature control of the SSAM at different stages in the mixing process is as follows [35]: the heating temperature of the mineral aggregate is 180–190 °C, the heating temperature of the modified asphalt is 165–170 °C, the mixing temperature is 165–170 °C, and the output temperature is 165–170 °C. The material temperature is 170 °C, the waste temperature is 195 °C, and the temperature on arrival at the construction site is 150 °C.

The journey from the mixing station to the paving site is about 30–40 min, and the transporter is covered with canvas for insulation. The laying of the AC-20 cushion was completed the day before the laying of the AC-16, and the top surface of the AC-20 was sprayed with tack coat oil.

- Paving and rolling

The receiving hopper of the paver is pre-painted with a release agent. The screed of the paver is preheated in advance and kept above 100 °C. The forward speed of the paver operation was maintained at 2 m/min.

On-site rolling is carried out in three stages: initial rolling, re-rolling, and final rolling using a double-drum roller and a tire roller. For the initial pressure, a double-drum roller was used for one static rolling and one vibration rolling at low speed. Double-drum and rubber rollers were used for cross-rolling in the re-rolling, and the rolling times were no less than four times. The final layer was rolled by a double-drum roller. To reduce the dissolution of snow-melting salts, a mop was used to apply light oil, such as soybean oil or rapeseed oil, on the steel wheel and rubber wheel. No water spray equipment was used throughout the rolling process.

- Active ingredient identification and pavement inspection

**Figure 9:**
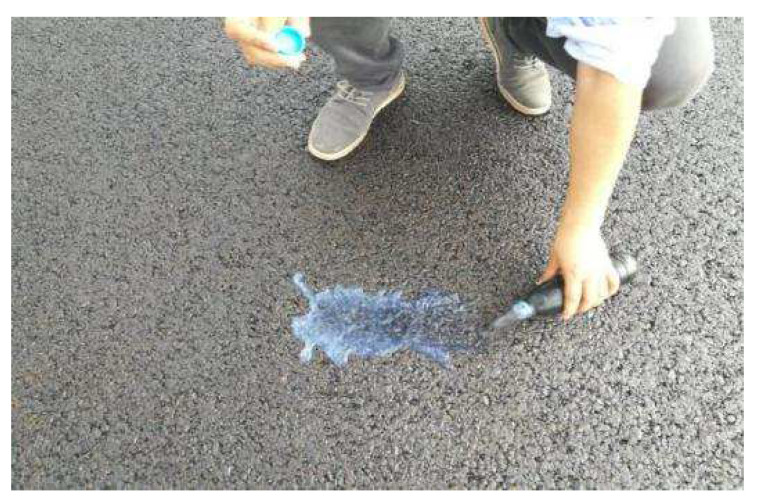
Active ingredient test of snow-melting salt.

The on-site confirmation method of the active ingredient of the slow-release snow-melting salt is the silver nitrate solution titration method. Pavements laid on site were tested for the presence of snow-melting salts at intervals using a formulated silver nitrate solution. The milky white color of the solution in Figure 9 proves that the snow-melting salt has withstood the high-temperature mixing and paving process. The existence of active ingredients proves that the experiment road can melt snow and ice in winter.

After the paving of the experiment road, the pavement was sampled by drilling cores. In addition, indexes, such as the permeability coefficient and flatness, were also tested. The test results are shown in Table 16. According to the test results, all the test indicators of the snow melting pavement meet the requirements of the specification; therefore, it is feasible to use the conventional hot mix asphalt pavement method for the salt-storage asphalt pavement.

## 4. Conclusions and Recommendations

(1) Snow-melting salt reduces the mechanical properties of SSAM. With the increase in the replacement amount of snow-melting salt, the mechanical properties of the asphalt mixture significantly decreased. Among them, the high-temperature performance of the SBS group SSAM decreased by 9.8–15.1%, the moisture resistance decreased by 6.3–19.4%, and the low-temperature performance decreased by 1.6–12.3%. All mechanical indexes of SBS50 meet the requirements, while the freeze–thaw splitting strength ratios of SBS75 and SBS100 do not meet the specification requirements.
(2) The mechanical properties of the HEA group SSAM with TPS are higher than those of the SBS group. Among them, the high-temperature, moisture resistance, and low-temperature performance of the SSAM in the HEA group are 11.3–19.7%, 4.8–13.3%, and 4.2–12.3% higher than those in the SBS group. This may be because the TPS not only improves the elasticity of SBS-modified asphalt but also improves the bonding performance between asphalt and aggregate. Therefore, the use of snow-melting salts with TPS is recommended.
(3) HEA can make up for the negative impact of snow-melting salt on the mechanical properties of SSAM to a certain extent, which makes the high-temperature performance, residual stability index of moisture resistance performance, and maximum bending tensile strain index of HEA50 and HEA75 not lower or even higher than SBS00 without snow-melting salt.
(4) In terms of surface properties, snow-melting salts can improve the skid resistance of SSAM, but they have a negative effect on surface spalling resistance. Whether it is a positive effect or a negative effect, the more snow-melting salt is added, the more obvious this effect is. Due to the high viscosity properties of HEA, HEA can both improve the anti-skid performance and surface anti-spalling performance.
(5) Considering both mechanical properties and surface properties, the comprehensive properties of the seven asphalt mixtures are HEA50, HEA75, SBS00, HEA100, SBS50, SBS75, and SBS100 from high to low. Therefore, HEA is suggested to be used as the cementing material of SSAM, and the content of snow-melting salt should be lower.
(6) In terms of engineering application, the mixture verified by indoor mechanics and surface properties can be used for salt-storage asphalt pavements. Moreover, the conventional paving method for hot asphalt mixtures can meet the acceptance requirements of salt-storage asphalt pavements.

In this paper, the mechanical properties, surface properties, and construction properties of SSAMs are studied from indoor and field perspectives. The durability such as fatigue performance of SSAM needs further exploration. It is worth noting that the comprehensive performance decision only considers the mechanical properties and surface properties, but in practical applications, the snow-melting salt content needs to be determined according to the snow-melting effect of the SSAM. In terms of on-site construction, the feeding method of snow-melting salt needs to be optimized.

## Figures and Tables

**Table 1:** Basic indicators of the two asphalts.

Index		Unit	SBS-Modified Asphalt	HEA	Standard	Test Method [31]
Penetration (100 g, 5 s, 25 °C)		0.1 mm	54	49	40–60	T0604-2011
Penetration index		-	0.55	0.68	≥0	T0604-2011
Ductility (5 °C, 5 cm/min)		cm	31	55	≥20	T0605-2011
Softening point		°C	80	94	≥60	T0606-2011
Kinematic viscosity at 135 °C		Pa·s	1.773	1.861	≤3	T0620-2000
Elastic recovery at 25 °C		%	90	98	≥75	T0662-2000
After TFOT	Quality loss	%	−0.213	−0.13	±1.0	T0610-2011
	Penetration ratio at 25 °C	%	70.5	76.6	≥65	T0604-2011
	Ductility (5 °C, 5 cm/min)	cm	16	31	≥15	T0605-2011

**Table 2:** Basic indicators of aggregates.

Aggregate	Index	Test	Standard	Test Method [32]
Coarse aggregate	Crush value (%)	10	≤26	T0316-2005
	Los Angeles attrition loss (%)	9.4	≤28	T0317-2005
	Firmness (%)	8	≤12	T0314-2000
	Soft stone content (%)	1.2	≤3	T0320-2000
	Needle-like content (%)	8.7	≤15	T0312-2005
Fine aggregate	Apparent specific gravity	2.663	≥2.5	T0328-2005
	Bulk specific gravity	2.731	/	T0330-2005
	Firmness (>0.3 mm) (%)	4	≤12	T0340-2005
	Sand equivalent (%)	70	≥60	T0334-2005

**Table 3:** Basic indicators of the two fillers.

Filler	Index	Test	Standard	Test Method [32]
Limestone	Moisture content (%)	0.2	≤1.0	T0332-2005
	Apparent specific gravity	2.762	/	T0352-2000
	Bulk specific gravity	2.667	/	T0352-2000
	Hydrophilic coefficient	0.73	<1	T0353-2000
	Plasticity index (%)	2.7	<4	T0354-2000
	Appearance	Qualified	No agglomeration	T0355-2000
Snow-melting salt	Appearance	White powder	No agglomeration	/
	Moisture content (%)	0.2	≤0.5	T0332-2005
	Apparent specific gravity	2.170	/	T0352-2000
	Bulk specific gravity	2.136	/	T0352-2000
	Salt content (%)	56	50 ± 10	/
	pH	8.3	8–8.5	/

**Table 4:** Technical indicators of lignin fibers.

Index	Ash Content/%	pH	Oil Absorption	Moisture Content/%	Width/mm	Bulk Density/(g/cm^3^)
Value	17	7.3	4–6 times the fiber mass	<5	<0.045	0.2–0.4

**Table 5:** Properties of SBS I-C for SSAM.

Index		Unit	Test	Standard
Penetration (100 g, 5 s, 25 °C)		0.1 mm	72	60–80
Penetration index		-	1.02	≥−0.4
Ductility (5 °C, 5 cm/min)		cm	51	≥30
Softening point		°C	82	≥55
Kinematic viscosity at 135 °C		Pa·s	1.763	≤3
Elastic recovery at 25 °C		%	92.5	≥65
After TFOT	Quality loss	%	−0.320	±1.0
	Penetration ratio at 25 °C	%	80.6	≥60
	Ductility (5 °C, 5 cm/min)	cm	30	≥20

**Table 6:** Density of aggregate and filler.

Particle Size/mm	0–3	3–6	6–11	11–17	17–22	Snow-Melting Salt
Apparent specific gravity/(g/cm^3^)	2.663	2.723	2.725	2.722	2.737	2.170
Bulk specific gravity/(g/cm^3^)	2.731	2.666	2.693	2.689	2.707	2.136

**Table 7:** Marshall test results.

Asphalt Aggregate Ratio/%	VFA/%	VV/%	Flow Value/mm	Stability/kN	Bulk Gravity/(g/cm^3^)
3.8	61.97	5.16	2.24	16.70	2.394
4.3	71.71	3.76	2.97	15.68	2.412
4.8	78.62	2.91	2.92	14.43	2.416
5.3	84.58	2.16	3.08	11.55	2.419
5.8	85.88	2.12	4.19	10.68	2.403
4.2 (optimum)	71.90	3.61	3.28	15.75	2.418

**Table 8:** Raw data for eight indicators.

SSAMs	Dynamic Stability/ (Times/mm)	Residual Stability Ratio/%	Freeze–Thaw Splitting Strength Ratio/%	Maximum Tensile Strain/με	Strain Energy Density/(J/m^3^)	BPN	Asphalt Loss/%	Cantabro Loss/%
SBS00	5258.4	93.9	89.4	2998.6	12,371.4	76	0.0560	5.7
SBS50	4744.9	88.0	82.3	2951.3	9531.6	79	0.0631	6.3
SBS75	4468.9	86.6	79.0	2910.8	9127.7	79	0.0670	7.5
SBS100	4462.1	86.4	72.1	2630.0	8448.5	81	0.0709	7.9
HEA50	5491.3	94.9	86.2	3149.8	13,912.6	83	0.0599	5.1
HEA75	5347.1	94.2	84.1	3033.4	13,246.8	85	0.0664	5.7
HEA100	4964.2	92.8	81.7	2954.7	11,204.5	88	0.0671	5.9

**Table 9:** Normalized data.

SSAMs	Dynamic Stability	Residual Stability Ratio	Freeze–Thaw Splitting Strength Ratio	Maximum Tensile Strain	Strain Energy Density	BPN	Asphalt Loss	Cantabro Loss
SBS00	0.958	0.989	1.000	0.952	0.889	0.864	1.000	1.000
SBS50	0.864	0.927	0.921	0.937	0.685	0.898	0.888	0.899
SBS75	0.814	0.912	0.884	0.924	0.656	0.898	0.835	0.757
SBS100	0.813	0.910	0.806	0.835	0.607	0.920	0.790	0.714
HEA50	1.000	1.000	0.965	1.000	1.000	0.943	0.935	1.119
HEA75	0.974	0.992	0.940	0.963	0.952	0.966	0.843	0.993
HEA100	0.904	0.978	0.914	0.938	0.805	1.000	0.834	0.966

**Table 10:** Comprehensive performance of SSAM.

SSAMs	Distances	Overall Performance Ranking
SBS00	0.187	3
SBS50	0.408	5
SBS75	0.526	6
SBS100	0.628	7
HEA50	0.151	1
HEA75	0.184	2
HEA100	0.296	4

**Table 11:** Dynamic stability of asphalt mixture.

Specimen Order	1	2	3	Mean	Standard
Dynamic stability/(times/mm)	6847.8	6326.7	5931.3	6368.6	1800–2800

**Table 12:** Marshall specimen residue stability.

Specimen Order	1	2	3	Mean	Standard
Stability before immersion/kN	15.86	15.92	16.69	/	/
Stability after immersion/kN	14.20	13.65	14.73	/	/
Residual stability ratio/%	89.56	85.71	88.25	87.84	≥80%

**Table 13:** Tensile strain at bending failure.

Specimen Order	1	2	3	Mean	Standard
Height/mm	35.5	35.4	35.2	/	/
Deflection/mm	0.4874	0.4991	0.5225	/	/
Span/mm	200	200	200	/	/
Tensile strain/με	2595	2650	2759	2668	≥2500

**Table 14:** Water permeability of SSAM.

Specimen Order	1	2	3	Mean	Standard
Water level at 1 min/mL	140	165	155	/	/
Water level at 2 min/mL	172	230	189	/	/
Water level at 3 min/mL	205	280	217	/	/
Permeability coefficient/(mL/min)	35	60	39	44.7	≤120

**Table 15:** The amount of materials required for one mixing.

1#silo	2#silo	3#silo	4#silo	5#silo	Snow- Melting Salt	Asphalt	Asphalt Aggregate Ratio	Total Aggregate
620 kg	355 kg	682 kg	630 kg	112 kg	100 kg	105 kg	4.2%	2499 kg

**Table 16:** On-site inspection results of salt-storage pavement.

Test Order	1	2	3	4	5	6	7	Design
Compactness/%	98.2	98.1	98.5	98.3	98.2	98.3	98.1	≥98
Permeability coefficient/(mL/min)	109	110	108	112	113	110	110	≤300
Flatness/mm	1.51	1.52	1.53	1.5	1.51	1.52	1.52	1.5

## Data Availability

All data used during the study appear in the published article.
